# Phosphatidylinositol metabolism of the renal proximal tubule S3 segment is disturbed in response to diabetes

**DOI:** 10.1038/s41598-023-33442-2

**Published:** 2023-04-17

**Authors:** Rosalie G. J. Rietjens, Gangqi Wang, Anouk I. M. van der Velden, Angela Koudijs, M. Cristina Avramut, Sander Kooijman, Patrick C. N. Rensen, Johan van der Vlag, Ton J. Rabelink, Bram Heijs, Bernard M. van den Berg

**Affiliations:** 1 Department of Internal Medicine (Nephrology), Leiden University Medical Center, Leiden, The Netherlands; 2 Einthoven Laboratory of Vascular and Regenerative Medicine, Leiden University Medical Center, Leiden, The Netherlands; 3 The Novo Nordisk Foundation Center for Stem Cell Medicine (reNEW), Leiden University Medical Center, Leiden, The Netherlands; 4 Department of Cell and Chemical Biology (Electron Microscopy), Leiden University Medical Center, Leiden, The Netherlands; 5 Department of Internal Medicine (Endocrinology), Leiden University Medical Center, Leiden, The Netherlands; 6 Department of Nephrology, Radboud University Medical Center, Nijmegen, The Netherlands; 7 Center for Proteomics and Metabolomics, Leiden University Medical Center, Leiden, The Netherlands

**Keywords:** Metabolomics, Diabetes, Kidney, Mass spectrometry

## Abstract

Diabetes is a main risk factor for kidney disease, causing diabetic nephropathy in close to half of all patients with diabetes. Metabolism has recently been identified to be decisive in cell fate decisions and repair. Here we used mass spectrometry imaging (MSI) to identify tissue specific metabolic dysregulation, in order to better understand early diabetes-induced metabolic changes of renal cell types. In our experimental diabetes mouse model, early glomerular glycocalyx barrier loss and systemic metabolic changes were observed. In addition, MSI targeted at small molecule metabolites and glycero(phospho)lipids exposed distinct changes upon diabetes in downstream nephron segments. Interestingly, the outer stripe of the outer medullar proximal tubular segment (PT_S3) demonstrated the most distinct response compared to other segments. Furthermore, phosphatidylinositol lipid metabolism was altered specifically in PT_S3, with one of the phosphatidylinositol fatty acid tails being exchanged from longer unsaturated fatty acids to shorter, more saturated fatty acids. In acute kidney injury, the PT_S3 segment and its metabolism are already recognized as important factors in kidney repair processes. The current study exposes early diabetes-induced changes in membrane lipid composition in this PT_S3 segment as a hitherto unrecognized culprit in the early renal response to diabetes.

## Introduction

Diabetes is one of the main risk factors for kidney disease, leading to diabetic nephropathy (DN) in close to half of all diabetic patients^1^. DN is characterized by a progressive, chronic loss of kidney function^2^. Hitherto, detection of albuminuria is the standard for diagnosis of DN, meaning that loss of glomerular function is already at play at time of diagnosis. This loss of glomerular function indicates that cellular changes have amassed as a consequence of the diabetic environment, leading to occurrence of structural and irreversible renal changes. There is a pressing need to better understand early cellular changes that occur upon diabetes, as this would open up a window of intervention before the structural integrity of the kidney is affected.

Matrix-assisted laser desorption/ionization mass spectrometry imaging (MALDI-MSI) has emerged as a powerful tool to investigate molecular characteristics of tissue^3^. In contrast to other mass spectrometry-based methods, there is no need to homogenize the tissue thus leaving the tissue architecture intact. MALDI-MSI is label-free and highly multiplexed, allowing spatial molecular characterization of the kidney at a resolution that facilitates cell type specific analysis^4,5^. The molecular profiles derived from MALDI-MSI analysis, in this case consisting of small molecule metabolites and glycero(phospho)lipids, can be used to visualize the metabolic histology of the kidney. This, in turn, can provide novel insights in molecular tissue heterogeneity, inaccessible using conventional histopathological analyses^6^.

The clinical effects of diabetes-related kidney disease can be predominantly allocated to resulting glomerulopathy, which explains the keen interest for glomerular research in this field. It is however not unlikely that other parts of the nephron, such as the renal tubules, are also affected by early changes in the microenvironment upon diabetes. Proximal tubule cells have been previously recognized as effectors in the progression of acute kidney injury (AKI) as well as chronic kidney disease (CKD)^7,8^. Furthermore, recent studies employing single cell RNA sequencing have shown various states of epithelial cell injury, all with their own distinct proinflammatory and profibrotic signature leading to differences in tubular repair outcomes after injury^9–11^. These differences in injury state have thus far not been directly linked to nephropathy caused by diabetes, though it is hypothesized that CKD might result in an increase in abundance of these cell states^12^. Therefore, the importance is evident to investigate molecular changes in nephron segments besides the glomerulus. Others have applied MALDI-MSI to investigate molecular changes of the kidney in animal models of early diabetes and DN^13–18^. These studies all revealed changes in renal metabolism upon diabetes, with differences in response between the cortical and medullary regions. However, variations in metabolic response between specific nephron segments have not been fully elucidated yet, underpinning the relevance of this work.

Here, we set out to identify early renal cell type specific changes in a mouse model of diabetes. MALDI-MSI-based metabolic histology of full kidney sections allowed us to gain insights into metabolic changes preceding irreversible structural renal changes following diabetes, especially those in the outer medullar proximal tubule segment.

## Results

### Streptozotocin-induced diabetes results in systemic metabolic changes

**Figure 1:**
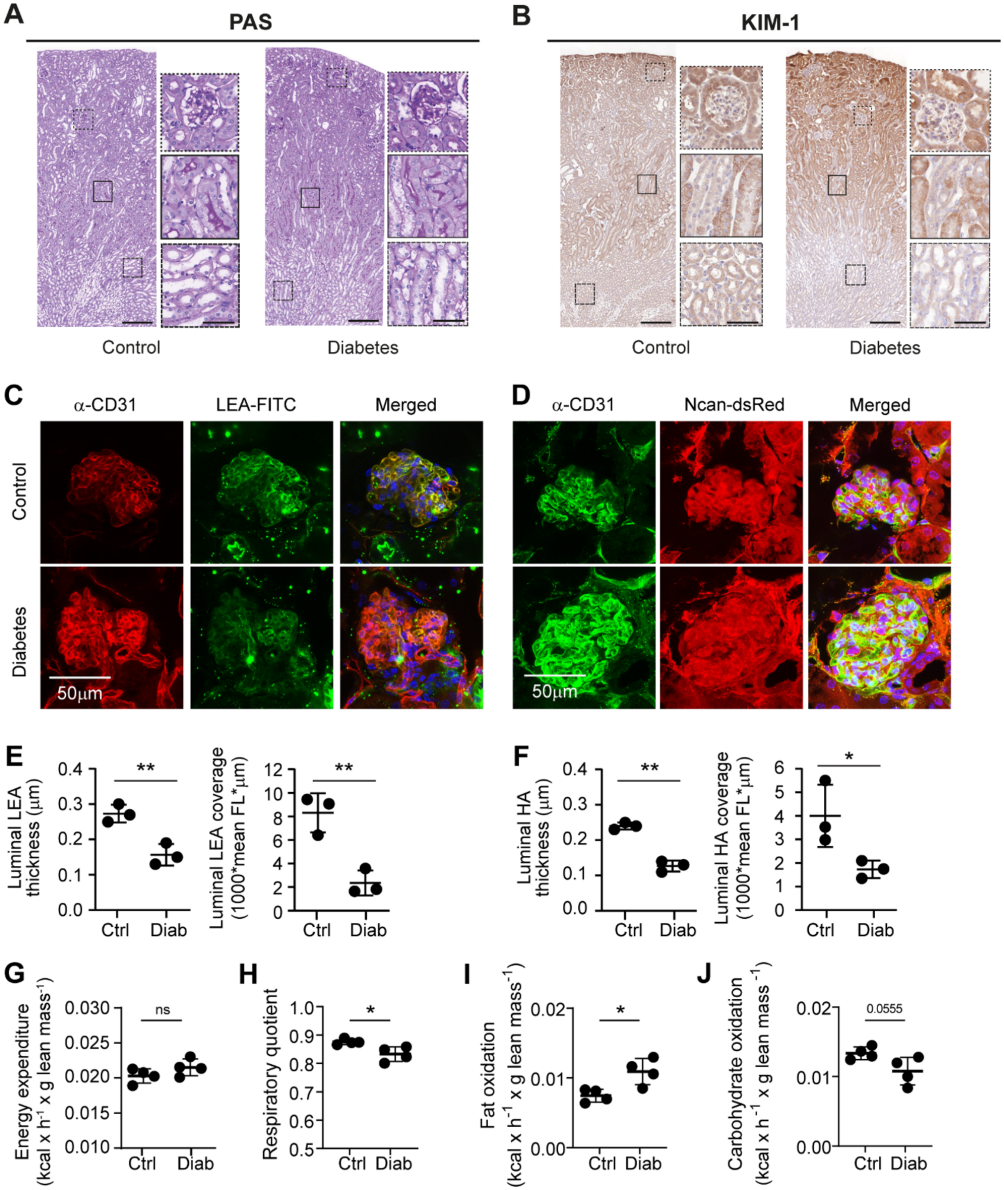
Diabetes in ApoE-KO mice manifests in systemic metabolic alterations without clear signs of DN. Representative images of control and diabetic mouse kidney sections stained with (**A**) periodic acid-Schiff (PAS) and (**B**) kidney injury molecule-1 (KIM-1), with insets showing more detailed images of the cortex, outer stripe of outer medulla (OSOM) and inner stripe of outer medulla (ISOM) (scale bar = 200 µm and for inset = 50 µm). Representative images of direct glycocalyx staining using fluorescent labeled (**C**) lectin *Lycopersicon esculentum* (LEA-FITC) and anti-CD31 antibodies for endothelial cell detection or (**D**) neurocan (Ncan-dsRed) and anti-CD31 antibodies; scale bar 50 μm. (**E** & **F**) Reduction of the luminal glycocalyx (LEA or Ncan-dsRed) thickness and luminal glycocalyx (LEA or Ncan-dsRed) coverage, assessed in a subset of *n* = 3 control and diabetic ApoE-KO mice. Systemic metabolic measurements comparing the (**G**) energy expenditure, (**H**) respiratory quotient, (**I**) fat oxidation rate and (**J**) carbohydrate oxidation rate at night of control and diabetic mice (*n *= 4/group used for subsequent MSI analysis). *HA* = *hyaluronan.*

To model diabetes-induced metabolic alterations of the kidney, we performed repeated injections of streptozotocin (STZ) in apolipoprotein E (ApoE) knock out mice (Supplementary Fig. 1A)^19,20^. At week 12, six weeks after the final STZ injection, diabetic mice manifested with hyperglycemia but without the presence of polyuria and albuminuria (Supplementary Fig. 1B–E). Pre-defined plasma glucose levels (15–20 mmol/L) were maintained by means of insulin administration when necessary. At the end of the experiment, 16 weeks after STZ induction, no apparent histologic changes indicative of DN were observed based on periodic acid-Schiff (PAS) staining, nor renal tubular injury was identified, using the kidney injury molecule-1 (KIM-1) marker (Fig. 1A,B). Glomerular surface and tuft area were not changed (data not shown), however early glomerular changes were observed by direct staining of PFA-fixed renal sections with the fluorescently labelled lectin *Lycopersicon esculentum* (LEA-FITC) or when stained specifically for hyaluronan (Ncan-dsRed) (Fig. 1C,D). Diabetes reduced the intraluminal lectin and hyaluronan thickness, and reduced coverage within the glomerular tuft which was further confirmed by visualizing glomerular intraluminal endothelial cationic ferritin coverage (Fig. 1E,F and Supplementary Fig. 1F). Combined, these results indicated that our model was representing early stage DN with merely minor glomerular changes. Testing systemic metabolism in these animals, using indirect calorimetric measurements at week 20, revealed that diabetic mice showed a similar energy expenditure but a significantly lower respiratory quotient during the dark phase, which was most likely explained by a switch from carbohydrate to fat oxidation (Fig. 1G–J). Day-time metabolism remained similar to control mice (data not shown). Given these early diabetes-induced changes we set out to analyze whether, despite the absence of clear histopathological hallmarks, metabolic changes in specifically the kidney had occurred. For this we used the MALDI-MSI in situ metabolomics approach.

### Metabolic histology and cell type identification in control and diabetic kidneys

**Figure 2:**
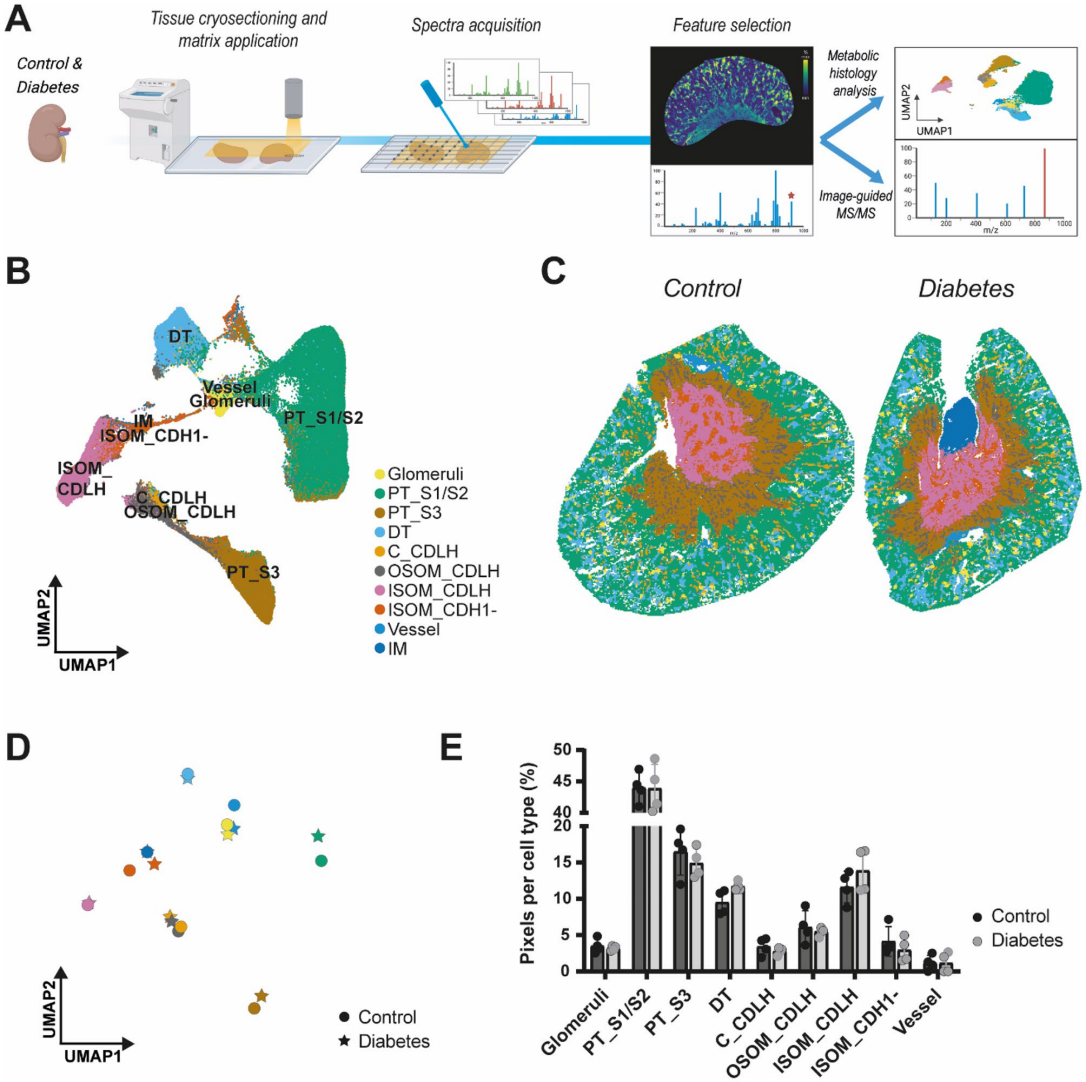
Metabolic histology of control and diabetes renal tissue. (**A**) Experimental workflow for in situ metabolic histology analysis of control and diabetic mouse. (**B**) Metabolic heterogeneity in the mouse kidney, visualized in a Uniform Manifold Approximation and Projection (UMAP) plot of MALDI-MSI data (*n* = 4/group). (**C**) Metabolic histology of control and diabetic renal tissue (example), identifying ten different renal cell types. (**D**) Visualization of the cluster centroids of the metabolic histological identified renal cell types, showing similarity between the control and diabetic renal clusters. (**E**) Relative contribution of the various renal cell types to the total pixel population in the control and diabetic mouse kidneys.

MALDI-MSI was applied to renal cryosections of control and diabetic mice (*n* = 4/group), providing information on small molecule polar metabolites and glycero(phospho)lipids. The in situ metabolomics data were used to perform metabolome-driven spatial segmentation analysis which resulted in various clusters with a high degree of molecular intra- and intergroup similarity (Fig. 2A, Supplementary Fig. 2A, B). For each of the clusters the unique marker ions (*m/z* features) were extracted revealing specific profiles per cluster (Supplementary Fig. 2C). Comparing the spatial distribution of these clusters and the corresponding metabolic markers with immunofluorescent (IF) staining of known renal cell type markers (*Lotus Tetragonolobus* lectin (LTL) for proximal tubular cells, *Bandeiraea simplicifolia* lectin (BS1) for endothelial/glomerular structures and antibodies against cadherin-1 (CDH1) for distal tubules, collecting duct and Loop of Henle; Supplementary Fig. 2D) allowed the annotation of clusters with specific renal cell types for both the control and diabetic mouse kidneys (Supplementary Fig. 3). This resulting metabolic histology revealed ten different renal cell types in both control and diabetic kidneys; glomerular, vascular, cortical proximal tubular segments (PT_S1/S2), the outer medullar outer stripe proximal tubular segment (PT_S3), distal tubular (DT), inner medulla (IM), CDH1 negative cells in the inner stripe of the outer medulla (ISOM_CDH1^-^), and collecting ducts and loop of Henle cells in the outer stripe of the outer medulla (OSOM_CDLH), in the inner stripe of the outer medulla (ISOM_CDLH) and in the cortex (C_CDLH) (Fig. 2B,C). The cluster centroid positions and the relative contribution of different cell types to the total cell population revealed merely minor differences between control and diabetes, indicating no large structural changes in metabolic histology upon diabetes (Fig. 2D,E). These results confirm that different cell populations in the kidney have distinct molecular signatures. It also indicates that, if present, the metabolic changes induced by early diabetes are more subtle compared to the inter cell type molecular variability. To address possible cell type specific differences, we performed nephron segment specific metabolic analysis to investigate the effect of diabetes on various segments of the kidney.

### Diabetes induces nephron segment specific metabolic changes

**Figure 3:**
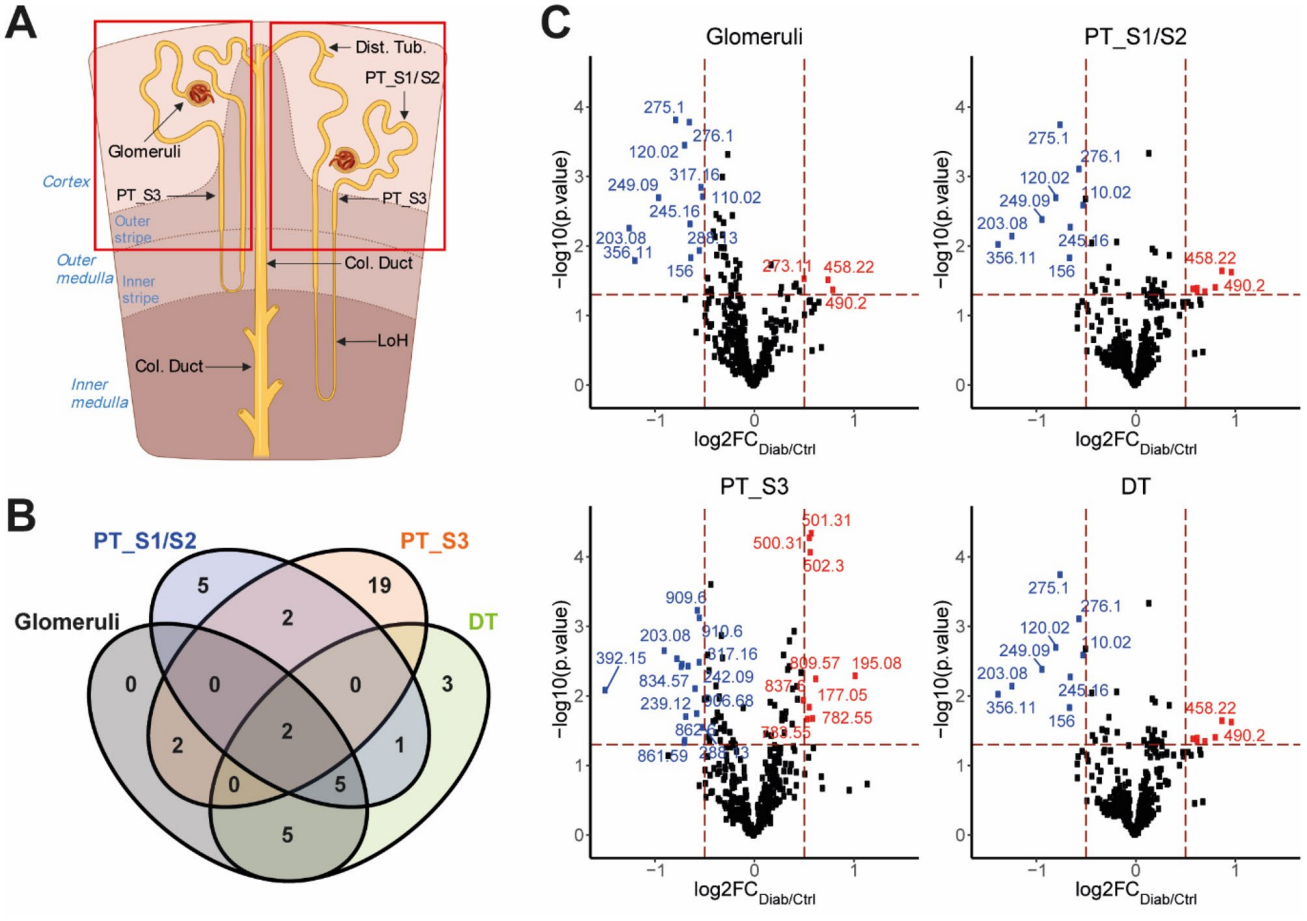
Changes in metabolic profile of specific renal cell types. (**A**) Schematic of a nephron, with the regions of interest for further data interrogation of metabolic characterization highlighted. (**B**) Venn diagram displaying the number of metabolites that are changed with a fold change larger or smaller than 0.5 or -0.5 respectively in the nephron segments. (**C**) Volcano plots of the nephron regions of interest, illustrating the changed metabolites in the glomeruli, PT_S1/S2, PT_S3 and DT of the diabetic mice compared to control.

To identify cell type specific changes in metabolites and/or lipids, univariate analyses of recorded metabolic features were performed comparing their abundance in specific renal cell segments between control and diabetic mice (Fig. 3A, Supplementary Table 1, 2, 3, 4). Since the glomeruli, proximal tubular- and distal tubular segments were unambiguously identified using the metabolic histology we decided to focus our analysis on these segments. Within these four segments, the number of unique differentially abundant metabolites was highest (19 features) in the outer stripe outer medullary proximal tubular segment (PT_S3) (Fig. 3B). Visualization of differential metabolites in these four cell clusters revealed that diabetes predominantly induced a reduction of metabolite abundance (Fig. 3C, Table 1). Only two metabolites (*m/z* 203.08 and 249.09) were altered consistently in the four segments between diabetes and control indicating heterogeneity between the segments in response to diabetes (Supplementary Fig. 4A). The decrease in these two metabolites, both assigned as products of protein catabolism, were indicative of altered renal protein processing in the diabetic mice. Furthermore, specifically in the lipid mass range (*m/z* ≥ 450) changes occurred primarily in the proximal tubular segments with most lipids being altered in the PT_S3 segment (Table 1). Hierarchical clustering on central carbon metabolite ratio fold changes between diabetic and control segments suggested PT_S3 responded differently to diabetes, as it clusters separately from the other segments (Supplementary Fig. 4B). Particularly the succinate to malate and glutamine to succinate ratios were changed in PT_S3 more pronounced compared to the other segments (-0.663 and 0.665, respectively). From this nephron segment specific metabolic analysis, we found that the PT_S3 segment displayed a unique response to diabetes in comparison to the other nephron segments.

**Table 1:** Metabolic features that are more (UP) or less (DOWN) abundant in glomerular, proximal tubular and distal tubular segments when comparing control and diabetic kidneys.

m/z	Glomeruli	PT_S1/S2	PT_S3	DT
110.0	DOWN	–	–	DOWN
120.0	DOWN	DOWN	–	DOWN
156.0	DOWN	–	–	DOWN
177.0	–	–	UP	–
195.1	–	–	UP	–
203.1	DOWN	DOWN	DOWN	DOWN
239.1	–	–	DOWN	–
242.1	–	–	DOWN	–
245.2	DOWN	–	–	DOWN
249.1	DOWN	DOWN	DOWN	DOWN
273.1	UP	UP	–	UP
275.1	DOWN	–	–	DOWN
276.1	DOWN	–	–	DOWN
288.1	DOWN	–	DOWN	–
302.2	–	–	UP	–
306.1	–	UP	–	UP
307.1	–	–	–	UP
308.1	–	–	–	UP
317.2	DOWN	–	DOWN	–
392.2	–	–	DOWN	–
356.1	DOWN	DOWN	–	DOWN
458.2	UP	UP	–	UP

	Glomeruli	PT_S1/S2	PT_S3	DT
465.3	–	UP	–	–
475.2	–	–	–	UP
490.2	UP	UP	–	UP
500.3	–	–	UP	–
501.3	–	–	UP	–
502.3	–	–	UP	–
721.5	–	DOWN	–	–
722.5	–	DOWN	–	–
782.6	–	–	UP	–
783.6	–	–	UP	–
786.6	–	–	DOWN	–
787.6	–	–	DOWN	–
809.6	–	–	UP	–
834.6	–	–	DOWN	–
837.6	–	–	UP	–
861.6	–	–	DOWN	–
862.6	–	–	DOWN	–
881.6	–	DOWN	–	–
906.7	–	–	DOWN	–
909.6	–	DOWN	DOWN	–
910.6	–	DOWN	DOWN	–
911.6	–	DOWN	–	–

### Fatty acid composition of phosphatidylinositol changes upon diabetes in PT_S3

**Figure 4:**
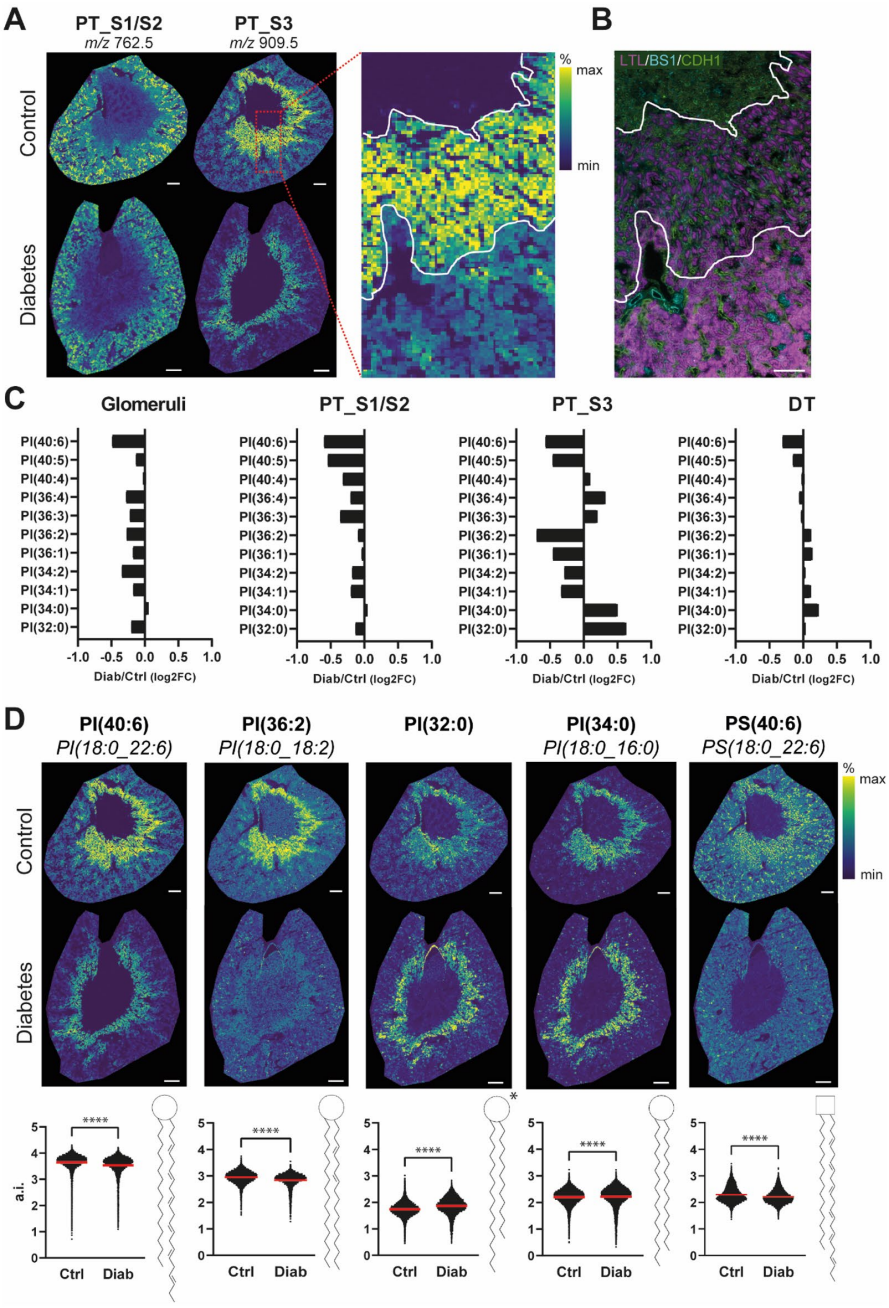
Lipid alterations in specifically PT_S3 cells upon diabetes. (**A**) Ion marker distribution of *m/z* 762.5 and *m/z* 909.5 clearly show the distinction between PT_S1/S2 and PT_S3, (**B**) confirmed by LTL^+^ staining and morphology. (**C**) Changes in phosphatidylinositol (PI) lipid species upon diabetes in the four nephron segments of interest: Glomeruli, PT_S1/S2, PT_S3 and DT. (**D**) In situ distribution and quantification of renal specific PS feature and PT_S3 specific PI features that are differentiating between diabetes and control. When tandem MS spectra are available, fatty acid tail composition is indicated and schematic drawings of the lipids are displayed (scale bar = 500 µm and for inset = 200 µm, *indicates author’s interpretation since tandem MS spectra were not available).

As mentioned above, lipid remodeling was restricted to the proximal tubular segments (Table 1). In the PT_S3 segment, seven lipid features were increased and eight were reduced. Further analysis using high mass resolution MALDI-FTICR-MSI provided putative lipid identity assignments (Table 2), and highlighted that the differentially abundant lipids in the PT_S3 segment between diabetes and control were members of the phosphatidylserine (PS) and phosphatidylinositol (PI) lipid classes. Spatial analysis revealed that this altered lipid profile could be allocated to the outer stripe outer medullary segment of the proximal tubules specifically and is not a general proximal tubule attribute (Fig. 4A,B). Additionally, differentially expressed PI lipids in the diabetic PT_S3 segment appeared to have shorter fatty acid chain length and higher degrees of saturation, which was not observed for the differentially expressed PS lipids (Fig. 4C, Supplementary Fig. 5). To get more in-depth information on the composition of the individual fatty acids—information not provided by the MALDI-FTICR-MSI measurements—we applied image-guided high-resolution tandem mass spectrometry (MALDI-FTICR-MS/MS) to lipids that clearly had an altered in situ distribution in control and diabetic kidneys (Fig. 4D and Supplementary Fig. 6). The distribution of distinctive lipid species showed that PI(18:0_18:2) (*m/z* 861.5462, ppm error = 4) and PI(18:0_22:6) (*m/z* 909.5466, ppm error = 4) were less abundant, specifically in the PT_S3 segment of the diabetic kidney, whereas PI(32:0) (*m/z* 809.5146, ppm error = 5) and PI(18:0_16:0) (*m/z* 837.5456, ppm error = 5) were more abundant (Fig. 4D). In contrast, PS(18:0_22:6) (*m/z* 834.5249, ppm error = 5) did not only display a segment specific reduction, but its intensity also appeared to be reduced throughout the entire kidney (Fig. 4D). In both the control and diabetes setting, stearic acid (18:0) remained to be incorporated as one of the side chains in the distinctive lipids. The second side chain however appeared to be affected by diabetes. In the diabetic PT_S3, PI species containing polyunsaturated fatty acids (e.g. 22:6 and 18:2) were less present in contrast to the control segment. On the contrary, saturated fatty acid side chains, typically with shorter carbon chain length (e.g. 16:0), were increased in the diabetic PT_S3 (Fig. 4D). Thus, it seemed that in the diabetic kidney the second side chain of PI lipid species was affected, where polyunsaturated fatty acids were exchanged for more saturated fatty acids with shorter chain length which was most pronounced in the PT_S3 segment (Fig. 4D). These results indicated that diabetes-induced alterations of PI lipid metabolism predominantly in the outer stripe outer medullary proximal tubules.

**Table 2:** More and less abundant lipids in PT_S3 cells. Asterix indicates the second lipid isotope.

m/z		Log2 foldchange	*p* value	Lipid annotation
786.6	DOWN	− 0.725	0.00378	PS(36:2)
834.6	DOWN	− 0.717	0.00350	PS(40:6)
861.6	DOWN	− 0.697	0.04685	PI(36:2)
862.6	DOWN	− 0.689	0.04332	PI(36:2)*
787.6	DOWN	− 0.656	0.00372	PS(36:2)*
906.7	DOWN	− 0.568	0.01787	–
909.6	DOWN	− 0.564	0.00059	PI(40:6)
910.6	DOWN	− 0.543	0.00075	PI(40:6)*
837.6	UP	0.502	0.01152	PI(34:0)
783.6	UP	0.542	0.02153	PS(36:4)*
500.3	UP	0.563	0.00005	LPE(20:4)
502.3	UP	0.571	0.00009	–
501.3	UP	0.582	0.00005	LPE(20:4)*
782.6	UP	0.596	0.02099	PS(36:4)
809.6	UP	0.626	0.00571	PI(32:0)

## Discussion

In the present study, we set out to identify possible early changes in renal metabolism in response to diabetes-induced systemic metabolic changes before overt pathophysiology was present. Using MALDI-MSI data of polar small metabolites and glycero(phospho)lipids, we were able to construct metabolic histology maps of control and diabetic kidney tissues. The metabolic and lipidomic profiles of the various metabolic cell types were further analyzed, highlighting the most pronounced changes in the outer stripe outer medullary proximal tubule segment PT_S3 compared to the other nephron segments. Lipid remodeling was restricted to the proximal tubule segments, with most features being altered in PT_S3. Of the various lipid classes identified with our MALDI approach, we found that especially the phosphatidylinositol metabolism in PT_S3 was disturbed upon diabetes. More specifically, the fatty acid tail composition of PI lipids was changed towards shorter chain length and higher degrees of saturation. Taken together, these results indicate that molecular alterations in the proximal tubular segment PT_S3 might be an early hallmark of diabetes-induced renal dysfunction.

Until now, the diagnosis of diabetes-induced renal changes is based on markers of glomerular function, the most prominent one being detection of albuminuria. Although effective in detecting a gradual decline of kidney function over time, these markers are present once structural changes of the kidney have already occurred^2^. As a result, at this point therapeutic strategies can merely be targeted at alleviation of symptoms and delay of further disease progression into kidney disease. It has been described previously that early diabetic nephropathy is accompanied by alterations in the kidney lipidome and metabolome, underlining the relevance of these omics to better understand early diabetes-induced changes that could eventually lead to diabetic nephropathy^21,22^. To date, MALDI-MSI has been proven effective to identify in situ metabolomic and lipidomic alterations of the kidney, however only in models presenting clear renal pathology^13,17^. The detection of nephron segment specific changes in a pre-clinical diabetic model as presented in this study provides new insights in the early commencement of diabetic kidney changes.

Previously, MALDI-MSI analysis has been targeted at either the total kidney—without sufficient spatial resolution to distinguish cell types—or only at the renal cortex^14–16^. These approaches, however, have overlooked the potential role of the proximal tubule S3 segment despite its known vulnerability in acute kidney injury^7,9,10^. Interestingly, in this study we revealed that specifically this PT_S3 segment has a unique metabolic response to diabetes compared to the cortical proximal tubule, glomerular and distal tubular segment. We recently showed that upon ischemia reperfusion injury the S3 segment displays an aberrant metabolic response compared to its convoluted counterpart^4^. For this we do argue that metabolic changes also affect this vulnerable segment and thereby possible priming it for injury by a second hit like known diabetic risk factors, e.g. hypertension.

Lipid remodeling in the PT_S3 as identified in this study might provide an explanation to the role of this segment in the progression of kidney disease. Aberrant lipid metabolism has been recognized as primary event and progression factor in diabetic kidney disease^22,23^. PI lipid species give rise to polyphosphoinositides which are important signaling molecules involved in, among others, cell proliferation, vesicle trafficking and apoptosis^24^. Despite being detectable by MALDI-MSI, the a priori selected mass range covered in this discovery study did not allow detection of these specific molecules. Although potentially relevant, we believe that the limited amount of changes in renal histopathology and functionality indicated little effect of direct changes in cellular signaling. On the contrary, we identified a trend in phosphatidylinositol—a major structural lipid found in cell membranes—fatty acid tail composition towards shorter chain length and higher degree of saturation. From studies focusing on membrane organization and stability, it is known that changes in balance between saturation levels of fatty acids in a membrane can greatly affect the rigidity and stability of that membrane^25,26^. Alterations of membrane stability and downstream signaling molecules could explain how early diabetes-induced alterations in PT_S3 phosphatidylinositol metabolism associate this segment with progression of diabetes-induced kidney injury.

In our experimental setup, both the control and diabetic group are from the ApoE-KO mouse strain, where diabetes is induced by STZ injections (citrate only in the control group) followed by a cholesterol-enriched diet. It has been shown that this model displays enriched plasma cholesterol levels compared to wild-type mice (≈400 mg/dL vs ≈80 mg/dL), regardless of diet^27^. Furthermore, in a different model of diabetes in the ApoE-mouse strain the levels of cholesterol in the diabetic mice were enriched, even in absence of a cholesterol-enriched diet^28^. Therefore, it is expected that in the current setup the cholesterol chow diet will not have had a significant effect on the cholesterol levels of mice. It is however interesting to consider whether the elevated cholesterol levels could have an effect on the PI metabolic changes in the PT_S3 segment. It has been shown that PI lipids can have an effect on cholesterol homeostasis, with PI promoting cholesterol transport and excretion^29^. When looking into the crosstalk between the metabolic pathways of PI lipids and cholesterol, it becomes apparent that these pathways are compartmentalized and linked through the common metabolite acetyl-CoA^30^. As glucose affects acetyl-CoA levels through altered rates of glycolysis, it is most likely that through the high glucose levels in the diabetic setting both cholesterol and PI lipid species are affected.

Limitations of the current study should also be addressed. Studies of renal proximal tubule functionality after induction of diabetes through STZ injection has been put to discussion previously^31^. At high doses (typically > 100 mg/kg), STZ is known to have direct cytotoxic effects and can cause renal tubular injury. In the current study we have however administered a low dose of STZ (60 mg/kg), without direct evidence of renal tubular injury. Therefore, the found alterations in the PT_S3 segment can most likely be attributed to the diabetic phenotype. A second limitation of the study is the lack of glomerular filtration rate (GFR) in this experimental setup. Although our results indicated lack of changes in glomerular functionality, additional direct measurements of the GFR would have been a valuable addition to confirm the integrity of glomeruli in the used model.

In conclusion, we found using metabolic histology analysis based on MALDI-MSI that the outer stripe outer medullary proximal tubule PT_S3 segment is sensitive to changes induced by diabetes. This previously overlooked proximal tubule segment might function as accelerating factor in diabetic nephropathy.

## Methods

### Diabetic ApoE-KO mouse model

Six-week-old male B6.129P2-*Apoe*^*tm1Unc*^/J mice (ApoE-KO; The Jackson Laboratory, Bar Harbor, ME) were rendered diabetic through intraperitoneal injections of 60 mg/kg streptozotocin (STZ; Sigma-Aldrich, St. Louis, MO) for 5 consecutive days, as described before^20^ (Fig. 1A). Control ApoE-KO mice were injected with vehicle alone and were used for baseline measurements. All mice had free access to standard rodent diet (NC; Sniff Spezialdiäten GmbH, Soest, Germany). Animals with persistent high blood glucose levels after two weeks were included in the study. At week 8, diabetic mice were fed cholesterol enriched (0.15%) chow until the end of the study. Blood glucose concentration by tail-tip blood droplets were measured with an Accu-check glucose meter (Roche, Basel, Switzerland). To overcome overt glucose toxicity mice were treated with 1–2 units insulin (Lantus, Aventis Pharmaceuticals, Bridgewater, NJ, US) when glucose concentrations exceeded 20 mmol/L. Mice were sacrificed at week 22 of age. All work with animals was performed in compliance with the ARRIVE guidelines^32^. Animal experiments were approved by the Ethical Committee on Animal Care and Experimentation of the Leiden University Medical Center (permit no. AVD1160020172926). All work with animals was performed in compliance with the Dutch government’s guidelines. For the present study a subset of control (*n* = 4) and diabetic (*n* = 4) mice were used for MALDI-MSI analysis of the total study (*n* = 9/group) as performed in the Dutch Kidney foundation/Health Holland consortium (GLYCOTREAT; grant number LSHM16058-SGF).

### Caloric measurements

In week 20, control and diabetic mice (total n = 9/group) were placed in metabolic cages (Sable Systems Europe GmbH, Berlin, Germany) for 4 days to measure food intake, oxygen consumption (V∙O_2_), carbon dioxide production (V∙CO_2_) and ambulatory physical activity (semi quantitative by beam breaks). Based on these values, the respiratory quotient (the ratio of carbon dioxide released to oxygen adsorbed), carbohydrate and fat oxidation rates were calculated to monitor the systemic metabolic state.

### Urine collection and analysis

After acclimatization in the metabolic cages (Tecniplast S.p.a., Buguggiate, Italy), 14 h-urine was collected at week 11, 17 and 21. Urine samples were centrifuged to remove debris and stored at -20 °C. Urinary albumin concentrations were quantified with an enzyme-linked immunosorbent assay (ELISA; Bethyl Laboratories, Inc. Montgomery, TX, USA) and creatinine concentrations were quantified by the Jaffe´ method using 0.13% picric acid and a creatinine standard set (Sigma-Aldrich, Merck Life Science NV, Amsterdam, The Netherlands).

### Tissue preparation and histology

Mice were anesthetized by isoflurane inhalation and subsequently perfused via the left ventricle with HEPES-buffered salt solution containing 0.5% bovine serum albumin and 5 U/mL heparin to remove blood. After removal of the kidney capsules, one kidney was placed in 2% PFA in PBS at 4 °C overnight, followed by paraffin embedding for staining. The left kidney of a subset of mice (n = 3/group) was perfused with 5 mL Hanks-buffered salt solution (HBSS, Gibco) containing 0.5% BSA (Sigma, A7030, essentially globulin free) and 5U/mL heparin at 2 mL/minute to remove blood, followed by 2 mL of cationic ferritin (horse spleen, 2.5 mg/mL, Electron Microscopy Sciences, Fort Washington, PA) in HBSS at 2 mL/minute. The kidney was stored in fixative (1.5% glutaraldehyde and 1% paraformaldehyde (PFA) (Electron Microscopy Sciences, Hatfield, PA) in 0.1 M sodium-cacodylate buffered solution, pH 7.4) overnight at 4 °C for further processing for transmission electron microscopy (TEM).

### Immunohistochemistry

Kidney Sects. (4 µm thick) were deparaffinized and washed with PBS, followed by antigen retrieval using target retrieval solution (Dako, Agilent Technologies, Vianen, The Netherlands). Endogenous peroxidases were blocked with 0.01% sodium azide and 1.5% hydrogen peroxide in PBS, subsequently sections were blocked with protein block serum free (Dako) for 1 h at room temperature (RT). Anti-mouse KIM1 antibody (5 µg/mL, R&D Systems, Abingdon, UK, MAB1817) was incubated overnight at 4 °C, followed by anti-rat HRP-labelled secondary antibody (Jackson Immunoresearch, Cambridgeshire, United Kingdom) for 1 h at RT. After incubation with DAB + substrate (Dako) for 12 min, hematoxylin (1:4) counterstain was performed, sections were rehydrated and mounted for imaging.

### Glomerular endothelial glycocalyx coverage

Glycocalyx coverage was determined using fluorescently labelled lectin *Lycopersicon esculentum* (LEA-FITC) and the N terminus rat neurocan construct of the hyaluronan specific neurocan-dsRed (Ncan-dsRed), as described previously^33,34^. In short, overnight PFA fixed tissue was sectioned in 100 μm thick slices with a Leica VT1000S vibratome (n = 3/group) and submerged in HBSS containing 0.5% BSA, 5 mmol/L HEPES, and 0.03 mmol/L EDTA (HBSS-BSA). Slices were incubated overnight at 4 °C on a shaker with 10 mg/mL LEA-FITC or Ncan-dsRed together with 5 mg/mL monoclonal mouse anti-mouse CD31 antibody (Santa Cruz Biotechnology, Santa Cruz, CA). After 3 washes with HBSS-BSA slices were incubated for 2 h at 4 °C on a shaker with 10 mg/mL Alexa Fluor-568, or AF488–conjugated goat anti-mouse IgG (Molecular Probes, Grand Island, NY) and Hoechst 33,528 (Sigma-Aldrich, 1:1000). Slices were fixated and mounted for imaging on a LEICA TCS SP8 X WLL microscope (Leica, Rijswijk, The Netherlands). Sequential 16-bit confocal images (xyz dimensions, 0.142 × 0.142 × 0.3 µm) were recorded using LAS-X Image software (Leica). The amount of endothelial glycocalyx was quantified using 5 lines of interest in 3–4 glomeruli per kidney by calculating the distance from the CD31 signal peak to the half-width of the luminal LEA-FITC or Ncan-dsRed signal along a line of interest, using intensity profiles (ImageJ), as described previously^34^.

### Sample preparation and MALDI-MSI measurement

Snap-frozen renal tissues of a subset of mice (*n* = 4/group) were embedded in deionized water and slices of 10 µm thickness were sectioned with a Cryostar NX70 cryostat at -20 °C. Indium-tin-oxide (ITO)-coated glass slides (VisionTek Systems Ltd., Chester, UK) were used to thaw-mount the sections. Subsequently, sections were placed in a vacuum freeze-dryer for 10 min. After drying, a total of 17 matrix layers with N-(1-naphthyl) ethylenediamine dihydrochloride (NEDC) (Sigma-Aldrich, UK) was applied. Tissues were measured by MALDI-MSI using a rapifleX MALDI-TOF/TOF system (Bruker Daltonics GmbH, Bremen, Germany). Negative ion-mode mass spectra were recorded covering a mass range from *m/z* 80—1 000 at a pixel size of 20 × 20 µm^2^ in reflectron mode. Spectra were recorded with 200 laser shots per pixel at a laser repetition rate of 10 kHz. Data was acquired using flexControl (Version 4.0, Bruker Daltonics, Germany) and data was visualized with flexImaging 5.0 (Bruker Daltonics). The same kidney samples were also measured on a 12 T solariX Fourier transform ion cyclotron resonance (FTICR) mass spectrometer to obtain MALDI-FTICR-MSI data for *m/z* annotation. Spectra were acquired in negative ion-mode, with 30 laser shots per pixel, using the Minimum laser focus setting at a pixel size of 50 × 50 µm^2^. Spectra were recorded covering a mass range from m/z 100–1,000 Th with a 512 k data point transient and transient length of 0.1049 s. Data was acquired using ftmsControl (Version 2.1.0, Bruker Daltonics), and data was visualized using flexImaging 5.0 (Bruker Daltonics). Both instruments were externally calibrated before measurements using red phosphorus.

### Tandem MS measurement and analysis

Image-guided MS/MS was performed on tissue sections prepared following the same procedure as for MALDI-MSI measurements. The 12 T solariX FTICR mass spectrometer was used to obtain MALDI-FTICR-CID-MS/MS data. Spectra were acquired averaging 10 scans with 150 laser shots, laser frequency 2 kHz and laser focus set at Minimum. Spectra were recorded over a mass range of *m/z* 245—1 000 Th with a 512 k data point transient and transient length of 0.3495 s. Collision energy was optimized per lipid (displayed in Supplementary Fig. 2). The LIPID MAPS product ion calculation tool^35^ for Glycerophospholipids was used to annotate the measured fragments.

### MALDI-MSI data preprocessing

The *m/z* features present in both MALDI-TOF-MSI and MALDI-FTICR-MSI datasets with similar tissue distributions were used to assign metabolites and lipid species. The *m/z* values from MALDI-FTICR-MSI were imported into the Human Metabolome Database (https://hmdb.ca/)^36^. Metabolite and lipid species annotation was performed with an error of ≤  ± 10 ppm. For small molecules only detected in MALDI-TOF, annotation was performed with an error of ≤  ± 20 ppm. MSI data were exported and processed in SCiLS Lab 2016b (Bruker Daltonics). All MALDI-TOF-MSI data was baseline corrected and normalized using the root mean square (RMS). Peaks from the average spectrum with a signal-to-noise-ratio higher than 3 (SNR ≥ 3) were selected and all matrix peaks were excluded. A total of 389 m*/z* features (containing small molecules and lipids) were selected by peak intensities for further data analysis.

### Post MALDI-MSI immunofluorescent staining

After MALDI-MSI measurements, the ITO glass slides were washed with 100% (2 × 5 min), 75% (1 × 5 min) and 50% (1 × 5 min) ethanol to remove remaining matrix. Tissue was fixed with 2% PFA in PBS for 15 min, blocked for an hour with 10% normal donkey serum and 0.3% Triton X-100 in PBS for 1 h at RT. Primary anti-E-Cadherin (1:300, BD Biosciences, Vianen, The Netherlands, 610,181), *Lotus Tetragonolobus* Lectin (LTL, 1:300, Vector Laboratories, Brunschwig Chemie, Amsterdam, The Netherlands, B-1325) or lectin from *Bandeiraea simplicifolia* isolectin B4 (BS-1-TRITC, 1:200, Sigma, L5264) were incubated overnight at 4 °C. When necessary, incubation with fluorescent-labeled secondary antibodies followed for 1 h at RT. Slides were mounted with ProlongTM gold antifade mountant with DAPI (Thermo Fisher Scientific, Amsterdam, The Netherlands, P36931). Slides were imaged using a 3D Histech Pannoramic MIDI Scanner (Sysmex, Etten-Leur, The Netherlands). Scanned fluorescent images were used for alignment to MALDI-MSI data.

### MALDI-MSI data analysis

Pre-processed MALDI-MSI datasets were transformed into a count matrix by multiplying intensities by 100 and taking the integer. SCTransform was used to normalize and scale the count matrix. UMAP analysis was performed on the count matrix using *Seurat 3.0* in R^37^, and the spatial projection of UMAP clusters on tissues were aligned to the IF staining. Cell type annotation was performed based on both staining and kidney morphology.

### Statistical analysis

Data are presented as mean ± SD unless indicated otherwise. Differences between groups were assessed by paired 2-tailed Student’s t test. *P*-value < 0.05 was considered statistically significant.

## Supplementary Information

Supplementary Information.

## Data Availability

The data underlying this article will be shared on reasonable request to the corresponding author.
