# Supplementary material for: Phosphatidylinositol metabolism of the renal proximal tubule S3 segment is disturbed in response to diabetes
Source: Sci Rep. 2023 Apr 17;13:6261. doi: 10.1038/s41598-023-33442-2 (PMC10110589; doi:10.1038/s41598-023-33442-2)
Supplement: Supplementary file 1 — Supplementary Information. [file 41598_2023_33442_MOESM1_ESM.docx]

**Supplementary material**


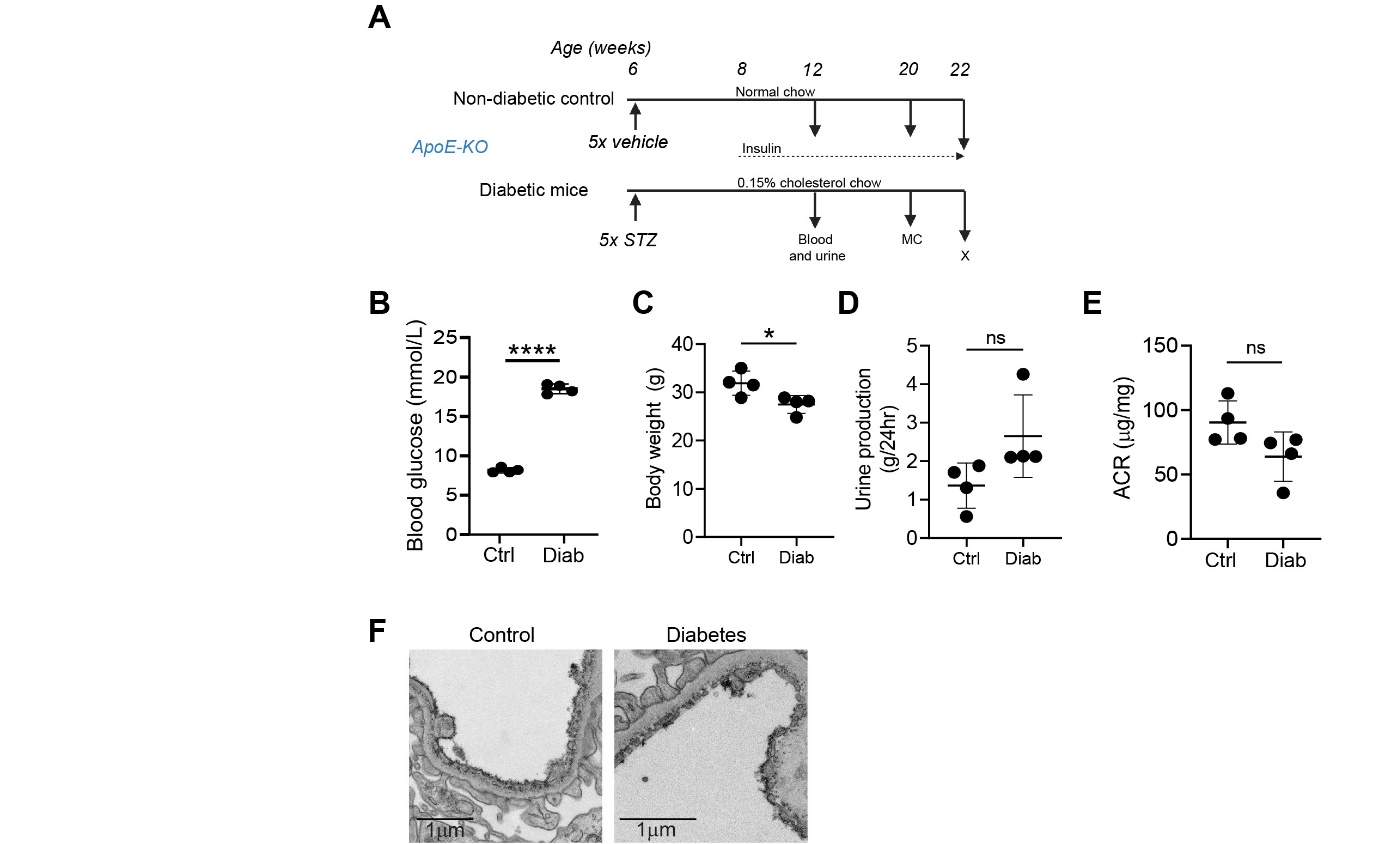


Supplementary Figure 1. Design of mouse study and baseline characteristics. (A) Schematic representation of the mouse study. Diabetes was induced in apolipoprotein E knock-out (ApoE-KO) mice by repetitive injection of streptozotocin (STZ), with the control group being repetitively injected with vehicle. Diabetic mice received a 0.15% cholesterol enriched chow diet. At 12 weeks, blood glucose levels were checked, at 20 weeks a subset of mice were put in a metabolic cage (MC) and at 22 weeks the mice were sacrificed. (B) Blood glucose levels, (C) body weight, (D) urine production and (E) albumin-to-creatinine ratio (ACR) of the control and diabetic mice used for mass spectrometry imaging analysis. (F) Representative transmission electron micrographs of cationic ferritin-stained glomerular endothelial surfaces in control- and diabetic ApoE-KO mice, performed as shown previously. (scale bar = 1 μm)


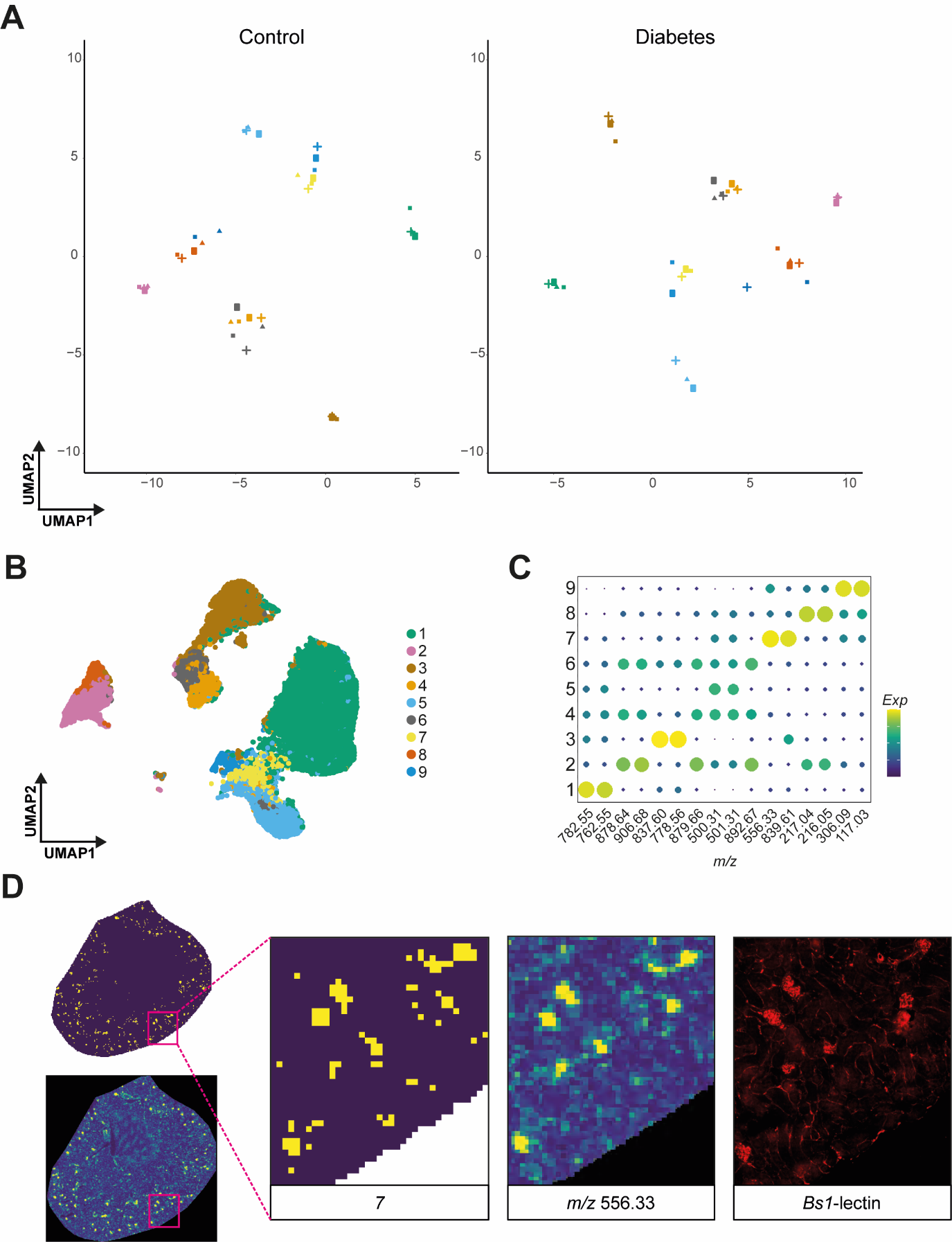


Supplementary Figure 2. Establishing the metabolic histology of the kidney. (A) Visualization of cluster centroids within the control and diabetic group, visualized in a uniform manifold approximation and projection (UMAP) plot revealing intragroup metabolic similarity per identified cluster. (B) Metabolic heterogeneity in control mouse kidneys (*n* = 4), visualized in a UMAP plot of MALDI-MSI data at 20 × 20 μm^2^ pixel size. (C) Dot plot displaying the metabolic markers of cluster-enriched features. (D) Workflow of immunofluorescence-assisted cluster annotation; overlaying the spatial distribution of cluster 7 with its metabolic marker and Bs-1 lectin staining allows annotation of the glomerular segment.


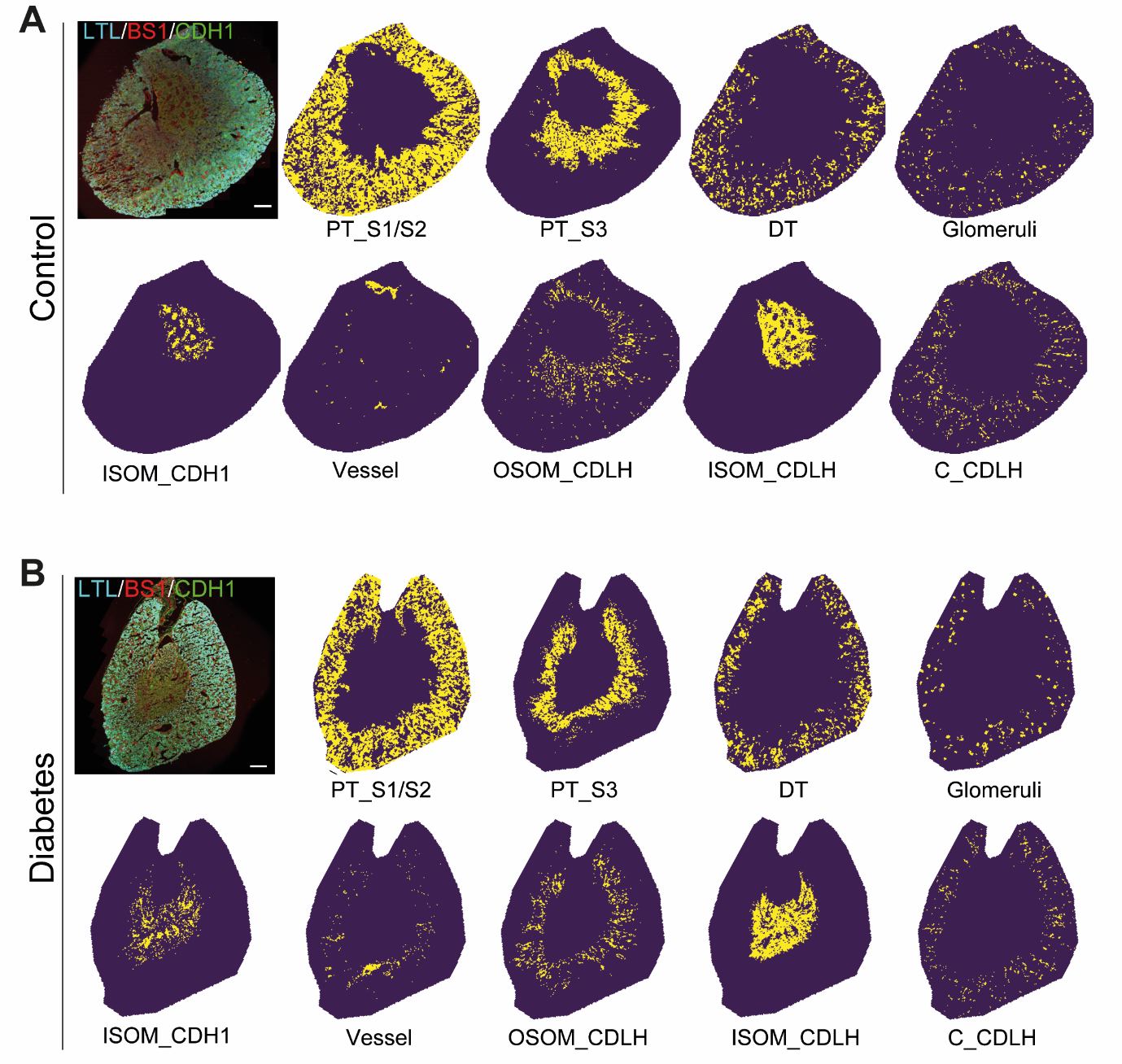


Supplementary Figure 3. Immunofluorescence-assisted cluster annotation. Post MALDI-MSI immunofluorescent staining (*Lotus Tetragonolobus* Lectin (LTL, blue), BS1-lectin (red) and
E-cadherin (CDH1, green)) was used to annotate the distribution of different renal cell clusters for the control (A) and diabetes (B) mouse kidneys (scale bar = 500 µm).


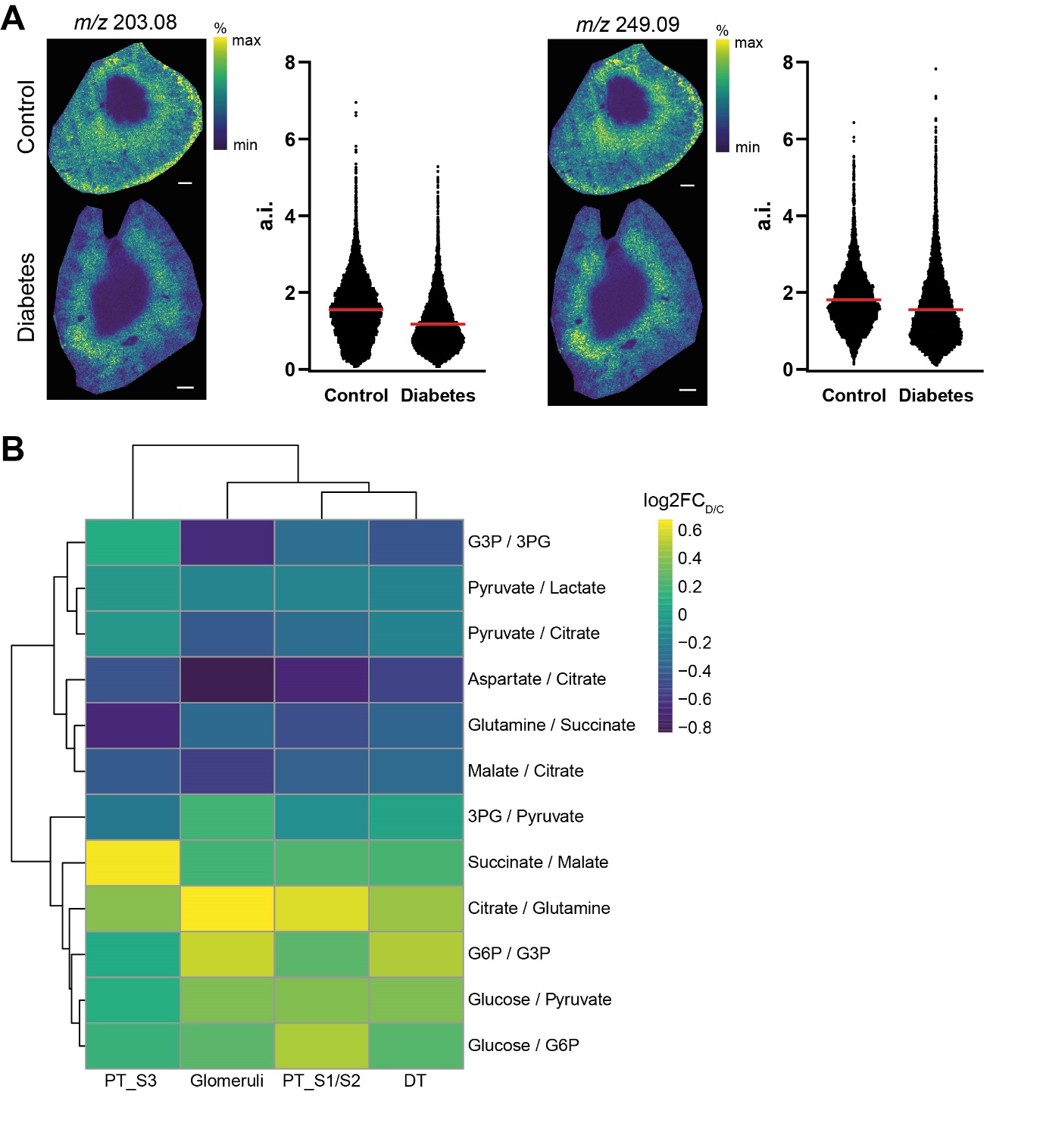


Supplementary Figure 4. Metabolic alterations of the kidney upon diabetes. (A) *In situ* visualization of *m/z* 203.08 and *m/z* 249.09, assigned to tryptophan (C_11_H_12_N_2_O_2_, ppm error = 13), and threonylmethionine (C_9_H_18_N_2_O_4_S, ppm error = 6) respectively. (B) Heatmap of log2FoldChange of metabolite ratios between diabetes and control per cell type.


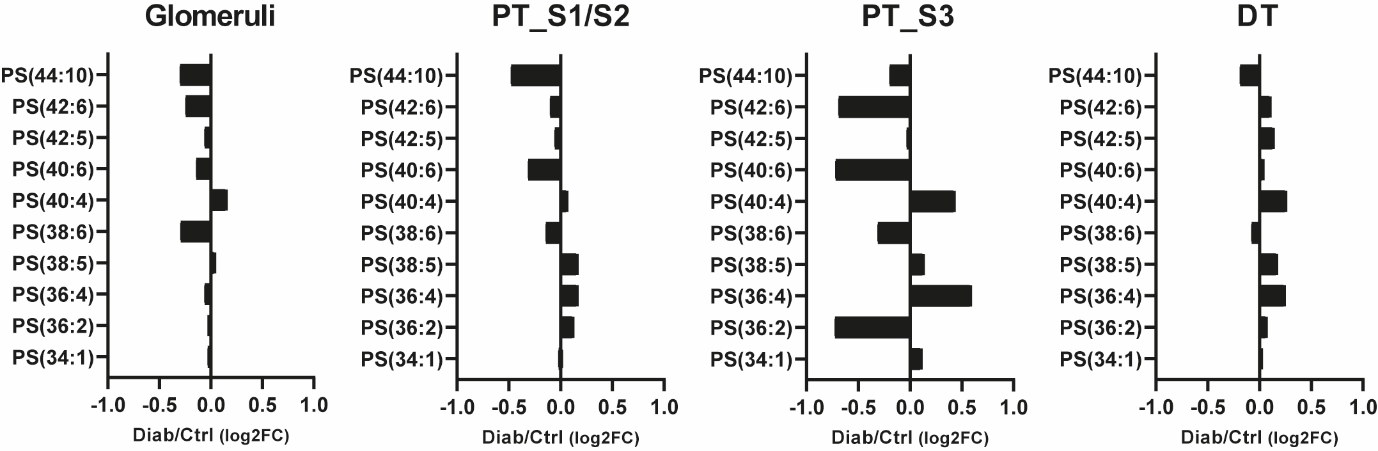


Supp Figure 5. Changes in PS lipid species upon diabetes in the full kidney, PT_S1/S2 and PT_S3 cells.


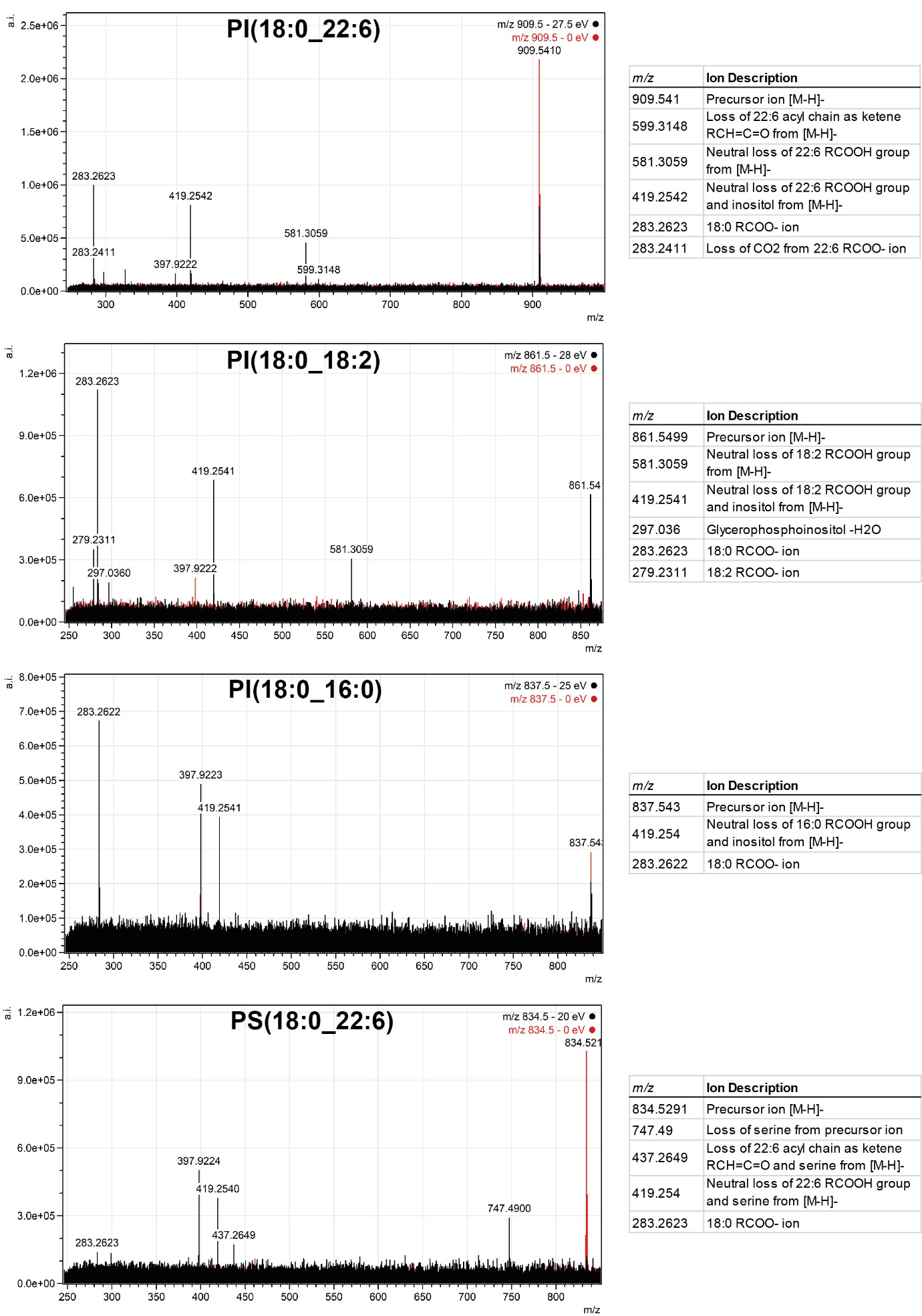


Supplementary Figure 6. Image-guided tandem mass spectrometry of distinctive lipid species. The red spectrum shows the pseudo-MS1 spectrum, where the lipid was isolated but no collision energy (0 eV) was applied. The black spectrum shows the MS/MS spectrum resulting from application of collision energy (optimized eV for each lipid). Due to isomerism, it cannot be determined which fatty acid occupies either of the two *sn*-positions.

**Supplementary Tables**

Table 1. Peak intensities of metabolic features that are more or less abundant in the glomerular segment of control and diabetic kidneys.

| Glomeruli | | | | | | | | |
| --- | --- | --- | --- | --- | --- | --- | --- | --- |
| Calibrated m/z value from MALDI-TOF | Peak intensity control (A.U.) | | | | Peak intensity diabetes (A.U.) | | | |
| 110.0 | 0.97 | 1.13 | 1.15 | 1.26 | 0.77 | 0.79 | 0.77 | 0.85 |
| 120.0 | 1.46 | 1.70 | 1.63 | 1.86 | 1.09 | 1.05 | 0.96 | 1.02 |
| 156.0 | 1.17 | 1.29 | 1.64 | 1.81 | 1.00 | 0.87 | 0.91 | 1.03 |
| 203.1 | 2.13 | 3.42 | 1.89 | 2.94 | 1.20 | 1.03 | 1.09 | 1.05 |
| 245.2 | 2.16 | 2.29 | 2.54 | 1.91 | 2.34 | 2.77 | 2.49 | 2.20 |
| 249.1 | 2.28 | 3.10 | 2.40 | 3.22 | 1.68 | 1.30 | 1.36 | 1.34 |
| 273.1 | 0.95 | 0.84 | 1.08 | 0.93 | 1.06 | 1.23 | 1.44 | 1.67 |
| 275.1 | 9.28 | 10.56 | 9.45 | 11.02 | 5.69 | 5.56 | 6.66 | 5.54 |
| 276.1 | 1.26 | 1.40 | 1.29 | 1.48 | 0.85 | 0.81 | 0.95 | 0.87 |
| 288.1 | 0.69 | 0.75 | 0.57 | 0.82 | 0.47 | 0.40 | 0.54 | 0.53 |
| 317.2 | 0.89 | 0.96 | 0.81 | 1.03 | 0.64 | 0.61 | 0.63 | 0.68 |
| 356.1 | 4.82 | 5.80 | 2.67 | 6.19 | 2.21 | 2.79 | 1.73 | 1.81 |
| 458.2 | 1.22 | 0.86 | 1.33 | 0.99 | 1.18 | 1.81 | 2.13 | 2.28 |
| 490.2 | 2.19 | 1.40 | 2.53 | 1.54 | 1.96 | 3.45 | 3.75 | 4.18 |

Table 2. Peak intensities of metabolic features that are more or less abundant in the proximal tubular S1/S2 segment of control and diabetic kidneys.

| PT_S1/S2 | | | | | | | | |
| --- | --- | --- | --- | --- | --- | --- | --- | --- |
| Calibrated m/z value from MALDI-TOF | Peak intensity control (A.U.) | | | | Peak intensity diabetes (A.U.) | | | |
| 120.0 | 0.93 | 1.08 | 0.98 | 1.29 | 0.77 | 0.62 | 0.73 | 0.69 |
| 203.1 | 1.57 | 2.45 | 1.48 | 2.17 | 0.99 | 0.78 | 0.99 | 0.92 |
| 249.1 | 1.65 | 2.04 | 1.65 | 2.36 | 1.23 | 0.87 | 1.21 | 1.04 |
| 273.1 | 0.53 | 0.47 | 0.60 | 0.56 | 0.64 | 0.61 | 0.86 | 1.00 |
| 306.1 | 15.77 | 8.87 | 17.49 | 10.28 | 14.36 | 21.93 | 28.02 | 30.81 |
| 356.1 | 3.69 | 4.18 | 2.29 | 4.44 | 1.89 | 2.19 | 1.44 | 1.60 |
| 458.2 | 0.61 | 0.47 | 0.71 | 0.57 | 0.70 | 0.85 | 1.19 | 1.31 |
| 465.3 | 1.02 | 0.96 | 1.12 | 0.90 | 1.39 | 1.45 | 1.68 | 1.25 |
| 490.2 | 0.98 | 0.63 | 1.17 | 0.76 | 1.08 | 1.55 | 2.04 | 2.28 |
| 721.5 | 2.78 | 2.67 | 1.60 | 2.51 | 1.34 | 1.15 | 1.09 | 1.12 |
| 722.5 | 1.47 | 1.47 | 1.04 | 1.34 | 0.90 | 0.79 | 0.87 | 0.83 |
| 881.6 | 6.79 | 6.05 | 4.83 | 4.69 | 3.74 | 4.47 | 3.65 | 3.79 |
| 909.6 | 17.13 | 16.08 | 12.91 | 11.79 | 9.60 | 10.78 | 8.81 | 9.14 |
| 910.6 | 9.32 | 8.74 | 7.01 | 6.43 | 5.28 | 5.89 | 4.82 | 5.03 |
| 911.6 | 4.83 | 4.56 | 4.46 | 3.67 | 2.94 | 3.09 | 3.19 | 2.81 |

Table 3. Peak intensities of metabolic features that are more or less abundant in the proximal tubular S3 segment of control and diabetic kidneys.

| PT_S3 | | | | | | | | |
| --- | --- | --- | --- | --- | --- | --- | --- | --- |
| Calibrated m/z value from MALDI-TOF | Peak intensity control (A.U.) | | | | Peak intensity diabetes (A.U.) | | | |
| 177.1 | 1.41 | 0.90 | 1.22 | 0.98 | 1.36 | 1.77 | 1.85 | 1.69 |
| 195.1 | 1.24 | 1.00 | 1.04 | 1.32 | 2.96 | 1.80 | 2.58 | 2.00 |
| 203.1 | 1.83 | 2.53 | 1.94 | 2.01 | 1.09 | 0.98 | 1.43 | 0.96 |
| 239.1 | 0.84 | 0.62 | 0.92 | 0.90 | 0.40 | 0.43 | 0.71 | 0.51 |
| 242.1 | 6.54 | 6.96 | 5.35 | 6.25 | 3.32 | 4.92 | 4.85 | 3.64 |
| 249.1 | 2.24 | 2.85 | 2.63 | 2.47 | 1.41 | 1.30 | 2.02 | 1.27 |
| 288.1 | 0.84 | 0.72 | 0.57 | 0.91 | 0.53 | 0.48 | 0.61 | 0.51 |
| 302.2 | 0.83 | 0.79 | 0.77 | 0.86 | 1.23 | 1.37 | 1.24 | 1.24 |
| 317.2 | 1.52 | 1.50 | 1.17 | 1.54 | 1.00 | 1.07 | 0.90 | 0.95 |
| 392.2 | 1.47 | 1.32 | 0.72 | 0.85 | 0.37 | 0.32 | 0.45 | 0.41 |
| 500.3 | 12.71 | 12.75 | 14.38 | 14.98 | 21.04 | 20.19 | 20.12 | 19.66 |
| 501.3 | 3.63 | 3.68 | 3.88 | 4.25 | 6.12 | 5.55 | 5.72 | 5.72 |
| 502.3 | 1.04 | 1.04 | 1.14 | 1.20 | 1.75 | 1.66 | 1.55 | 1.61 |
| 782.6 | 1.97 | 1.73 | 2.41 | 1.78 | 3.24 | 3.49 | 2.17 | 3.03 |
| 783.6 | 0.99 | 0.87 | 1.21 | 0.93 | 1.57 | 1.69 | 1.10 | 1.47 |
| 786.6 | 4.71 | 4.39 | 3.44 | 3.54 | 2.55 | 2.59 | 1.99 | 2.59 |
| 787.6 | 2.26 | 2.11 | 1.67 | 1.74 | 1.28 | 1.29 | 1.05 | 1.32 |
| 809.6 | 4.96 | 3.72 | 3.63 | 3.77 | 6.72 | 7.11 | 5.49 | 5.51 |
| 834.6 | 9.47 | 9.61 | 10.83 | 7.32 | 5.92 | 5.89 | 4.81 | 6.02 |
| 837.6 | 8.77 | 7.52 | 6.05 | 6.80 | 10.92 | 11.73 | 9.55 | 9.05 |
| 861.6 | 19.12 | 17.41 | 9.41 | 13.06 | 9.73 | 10.34 | 7.72 | 8.60 |
| 862.6 | 9.85 | 9.06 | 5.06 | 6.70 | 5.08 | 5.41 | 4.01 | 4.53 |
| 906.7 | 0.82 | 0.79 | 0.52 | 0.65 | 0.47 | 0.51 | 0.46 | 0.43 |
| 909.6 | 39.57 | 39.74 | 36.46 | 32.98 | 25.97 | 27.25 | 24.16 | 23.25 |
| 910.6 | 21.48 | 21.52 | 19.70 | 17.84 | 14.21 | 15.02 | 13.14 | 12.89 |

Table 4. Peak intensities of metabolic features that are more or less abundant in the distal tubular segment of control and diabetic kidneys.

| DT | | | | | | | | |
| --- | --- | --- | --- | --- | --- | --- | --- | --- |
| Calibrated m/z value from MALDI-TOF | Peak intensity control (A.U.) | | | | Peak intensity diabetes (A.U.) | | | |
| 110.0 | 0.68 | 0.79 | 0.78 | 0.87 | 0.58 | 0.47 | 0.53 | 0.59 |
| 120.0 | 0.90 | 1.09 | 0.94 | 1.20 | 0.72 | 0.51 | 0.54 | 0.61 |
| 156.0 | 0.74 | 0.86 | 0.96 | 1.15 | 0.69 | 0.46 | 0.55 | 0.65 |
| 203.1 | 1.49 | 2.38 | 1.27 | 2.00 | 0.91 | 0.64 | 0.72 | 0.77 |
| 245.2 | 0.73 | 0.67 | 0.55 | 0.80 | 0.47 | 0.39 | 0.42 | 0.48 |
| 249.1 | 1.53 | 2.03 | 1.48 | 2.13 | 1.19 | 0.76 | 0.88 | 0.93 |
| 273.1 | 0.97 | 1.19 | 0.56 | 0.61 | 0.94 | 0.60 | 0.49 | 0.63 |
| 275.1 | 6.54 | 6.86 | 6.00 | 7.44 | 4.20 | 3.61 | 4.20 | 3.94 |
| 276.1 | 0.88 | 0.94 | 0.86 | 1.03 | 0.66 | 0.54 | 0.65 | 0.67 |
| 306.1 | 21.05 | 13.32 | 26.94 | 14.73 | 23.27 | 29.24 | 37.13 | 43.76 |
| 307.1 | 2.91 | 2.04 | 3.73 | 2.17 | 3.21 | 3.89 | 4.81 | 5.76 |
| 308.1 | 1.30 | 0.98 | 1.61 | 1.09 | 1.48 | 1.66 | 2.06 | 2.53 |
| 356.1 | 3.03 | 3.85 | 1.68 | 3.73 | 1.37 | 1.22 | 1.07 | 1.08 |
| 458.2 | 0.76 | 0.54 | 0.88 | 0.67 | 0.94 | 1.07 | 1.50 | 1.72 |
| 475.2 | 0.54 | 0.47 | 0.73 | 0.53 | 0.70 | 0.72 | 0.93 | 1.15 |
| 490.2 | 1.32 | 0.82 | 1.63 | 0.99 | 1.54 | 2.08 | 2.65 | 3.08 |
